# Integrative Gene-Centric Analysis Reveals Cellular Pathways Associated with Heritable Breast Cancer Predisposition

**DOI:** 10.3390/cancers17243969

**Published:** 2025-12-12

**Authors:** Roei Zucker, Shirel Schreiber, Amos Stern, Michal Linial

**Affiliations:** 1 The Rachel and Selim Benin School of Computer Science and Engineering, The Hebrew University of Jerusalem, Jerusalem 9190401, Israelamos.stern@mail.huji.ac.il (A.S.); 2 Department of Biological Chemistry, The Life Science Institute, The Hebrew University of Jerusalem, Jerusalem 9190401, Israel; shirel.schreiber@mail.huji.ac.il

**Keywords:** UK Biobank, FinnGen, PWAS, MVP, rare variants, GWAS, Phecode, population structure, bioinformatics

## Abstract

**Background:** Heritable breast cancer (BC) predisposition is strongly influenced by high-penetrance genes such as BRCA1 and BRCA2, but many moderate- and low-penetrance genes remain poorly characterized. Although over 100 loci have been reported, the causal genes often include false positives or uncertain associations. **Methods:** We applied a gene-centric, integrative approach to multi-ethnic genomic datasets, including the UK Biobank (UKB) and FinnGen (FG). We assessed consistency across multiple GWAS in Open Targets (OT) and additional complementary genetic association approaches, including ExPheWAS, TWAS, and PWAS. Collapsing variant-level effects to a gene-level view enhanced confidence and reaffirmed contributions from genes such as BRCA1, BRCA2, PALB2, CHEK2, and other DNA repair genes. **Results:** Using this integrative framework, we identified 38 high-confidence BC predisposition genes, including 8 previously reported drivers, 13 supported by multiple lines of evidence, and additional candidates (e.g., APOBEC3A, TNS1, PEX14) with emerging evidence. PWAS revealed several genes with potential recessive effects often missed by standard GWAS. Multi-cohort replication showed robust findings in European ancestry populations, while transferability to other populations was more limited. **Conclusions:** This work demonstrates the value of a gene-centric, integrative framework for prioritizing high-confidence BC predisposition genes, highlighting associated cellular pathways, and uncovering new candidates for further functional study, providing a reliable foundation for future research.

## Simple Summary

Heritable breast cancer (BC) is influenced by well-known high-penetrance genes such as BRCA1, BRCA2, PALB2, and CHEK2, but the contribution of many moderate- and low-penetrance genes remains unclear. This study used large, multi-ethnic genomic datasets, including the UK Biobank and FinnGen, to refine the set of genes associated with BC predisposition. By integrating multiple association approaches (GWAS, TWAS, and PWAS) and removing likely false positives, we reported on 38 high-confidence BC predisposition genes. These include established DNA repair genes and new candidate genes supported by independent evidence. PWAS also highlighted genes with potential recessive effects that are often missed by standard GWAS. Findings were most consistent in European ancestry populations. Overall, this work provides a conservative, gene-focused framework to clarify biological pathways and prioritize candidate genes for further functional study.

## 1. Introduction

Breast cancer (BC) is one of the most common malignancies globally, with its development driven by both genetic predisposition and environmental/lifestyle factors like age, obesity, and hormonal exposure [1,2,3,4,5]. A strong family history of BC is a significant risk factor, highlighting the role of shared genetics and environment [6].

Approximately 5–10% of all BC cases are associated with hereditary genetic mutations [7]. The most well-known high-penetrance tumor suppressor genes, *BRCA1* and *BRCA2*, were identified through family-based linkage studies and are among the most significant BC predisposition genes [8]. The impact of these mutations on cancer risk, particularly for BC, differs between males and females [9]. While *BRCA1/2* are clinically important, their overall contribution at the population level is modest. Other high-penetrance genes, such as *TP53*, *RAD51C*, *RAD51D*, and *PALB2*, also markedly increase lifetime risk [10,11]. Additionally, polygenic risk scores (PRS) have helped identify moderate-to-low penetrance genes like *CHEK2*, *ATM*, *PTEN*, and *PPM1D* [12]. The integration of next-generation sequencing (NGS) technologies in clinical oncology now allows for more precise individual risk assessments, especially in specific genetic subpopulations [13,14,15].

Despite the growing body of genetic data, there is no consensus on a definitive set of genes that govern BC genetic risk, and their general applicability across diverse populations remains unclear. While some candidate predisposition genes are included in testing panels, treatment and therapy strategies based on these findings are not yet conclusive [16,17,18]. The field has been shaped by numerous associated variants from Genome-Wide Association Studies (GWAS). To date, about 200 independent loci have been linked to BC susceptibility, though most were identified at sub-genome-wide significance thresholds (e.g., *p* < 1.0 × 10^−6^). These loci generally have small or negligible effect sizes (odds ratios < |1.1|), but their cumulative impact has been used in PRS for risk stratification at the population level [19], and specifically for carriers of high-penetrance variants [20]. The importance of adding PRS for refining clinical stratification was confirmed [21].

Cross-cohort replication studies have further refined our understanding of population-specific risk architectures. For example, the Finnish genetic project (FinnGen, FG) [22] has uncovered rare, population-enriched variants in known BC genes (*BRCA1/2*, *RAD51C*, *BARD1*) due to founder effects [23]. Similar founder mutations in *CHEK2* (1100delC) and *PALB2* have been identified, with similar genes found in Korean women [24]. These large-scale genetic resources validate known risk genes and identify novel, population-specific variants, underscoring the roles of evolutionary forces like migration and genetic drift [25]. A notable example is a large *BRCA1* deletion (exons 9–12), a founder mutation among women of Mexican descent that contributes to a high rate of hereditary BC [26]. Three specific founder frameshift mutations, *BRCA1* (185delAG, 5382insC) and *BRCA2* (6174delT), are approximately 10-fold more prevalent in populations of Ashkenazi Jews in Israel compared to the general population [27]. High rates of consanguinity can further increase the frequency of pathogenic germ-line variants by reinforcing founder effects and reducing genetic diversity, as documented in Palestinian women with unusual prevalence *TP53* predisposition [28]. These patterns illustrate that inherited BC risk is shaped by both individual gene effects and broader population genetic structures.

The UK Biobank (UKB) is a large resource of 500,000 participants, predominantly of European descent [29]. In the UKB, pathogenic *BRCA1/2* variants are found in less than 0.5% of women, and most BC cases occur in individuals without any known pathogenic mutations. The hereditary component of triple-negative BC (TNBC), which has a poor prognosis, shares features with *BRCA1*-deficient tumors. Approximately 70 loci have been identified through GWAS and large-scale replication studies [30]. A conservative list of about 20 predisposition genes can be categorized by their functions in cell cycle, DNA repair, adhesion, and endothelial physiology [31]. Understanding the distribution of these mutations across different ethnic and geographic groups is crucial for effective screening, genetic counseling, and equitable access to precision medicine [25,32].

This study addresses several understudied aspects of BC predisposition genetics. Our analysis emphasizes the identification of susceptibility genes rather than isolated causal variants. First, we revisited the list of GWAS credible genes by leveraging external resources such as the Open Targets (OT) gene-association platform [33] to prioritize genes based on both genetic associations and complementary non-genetic evidence. The analysis spans a spectrum of genetic effects, from high-penetrance variants in *BRCA2* to common variants with modest risk contributions. Second, we re-examined large biobank datasets (UKB, FG, and MVP) to test gene-level coherence and cross-cohort agreement in the discovery of genes with low to moderate effect sizes. Finally, we reanalyzed the data using multiple complementary genetic association approaches: PWAS for protein-coding variants, TWAS for expression-altering variants, and several GWAS sets, to derive a high-confidence core list of BC susceptibility genes. This integrative gene-centric framework strengthens our understanding of the genetic architecture of BC predisposition and lays the groundwork for functional exploration of these genes and their pathways. Ultimately, this research supports the translation of molecular insights into clinically actionable strategies for improved BC risk assessment and targeted prevention.

## 2. Materials and Methods

### 2.1. Biobank Data Processing

The UK Biobank (UKB) is a population-scale resource that integrates deep phenotypic, genotypic and lifestyle information on roughly 500,000 volunteers aged 40–69 recruited across the United Kingdom between 2006 and 2010 with follow-up and ongoing update of diagnosis (2024-Q3 data release). For genetic analysis, we removed related individuals by keeping randomly chosen participant per kinship cluster [34]. In the PWAS results, the samples were further restricted to genetically inferred Europeans (UKB data-coding 1, 1001, 1002, 1003, and field 21000). A complementary, whole-cohort view retains the ~78,000 participants labeled “non-white-British”. We adhere to the definition from Phecode 174.11 (Supplementary Figure S1) based on mapping to ICD-10 (International Statistical Classification of Diseases and Related Health Problems,10th Revision) indexed C50; UKB field 41270 [35]. This study uses the UKB application ID 26664 (Linial lab).

FinnGen Freeze 12 (November 2024, FG12) provides >21.2 M variants (deep sequencing enriched) from >500,000 Finnish individuals (median age 63 y, 56.4% women). Standard quality filters removed variants with >2% missingness, HWE *p* < 1 × 10^−6^ or MAC < 3. All FG12 analyses adjusted for sex, age, 10 PCs, chip version and batch. We analyzed the results from the cohort of C3_BREAST_EXALLC with 24,270 cases and 222,078 controls. In addition, we tested subtypes of breast cancer (C3) marked as C3_BREAST-ERNEG_EXALLC (9724 cases) and C3_BREAST-ERPLUS_EXALLC (14,540 cases). There are 221,705 controls in FG12. We reanalyzed the GWAS fine-mapping with Bayes factors, yielding high-confidence variants. We also analyzed the coding-variant subset and the PheWAS interrogation activated by Ristey R13 (v2.7.1) query interface [22]. FG12 was considered a disjoint cohort and used for replication of gene discovery and increasing confidence

In this study, we re-analyzed the FG12 fine-mapping results using the FINEMAP v1.4 framework. The analysis applied FinnGen’s LD matrices together with the standard Bayesian settings, including a prior causal SNV probability of 1 × 10^−4^, ≥10 causal variants per locus, and the assumption of approximately independent LD blocks. Credible sets (CS) were defined using the posterior inclusion probabilities (PIP) provided by FG, which quantify the probability that each variant is causal. Variants were ranked by PIP, and the smallest set whose cumulative PIP reached 95% was reported as the 95% CS.

### 2.2. GWAS, Coding-Gene GWAS and Summary Statistics Analyses

The data from the UKB was analyzed as described before [36]. We performed GWAS in two versions, the routine GWAS and the gene-length GWAS (cgGWAS). Among the 487,409 participants with inpatient records (field 41270, September 2024), 16,952 carried at ICD-10 C50 code, 64% among them were diagnosed upon hospital admission. The GWAS-for C50 comprised 16,952 cases and 409,651 controls with genotyping imputed data. GWAS was based on the genotyping data with approximately 820k chosen genetic variations available for every participant. We employed a standard PLINK ver. 2.00a2.3LM (64-bit Intel architecture; release date: 24 January 2020) procedure for the analysis. The genotyping information is based on the UKB Axiome Array. We applied UKB imputation protocol and the count 97,013,422 variants. For the imputed variants, we computed the probabilistic expectations for the alternative alleles [37]. We have conducted analysis that is based on coding gene length (cgGWAS) using the MAF threshold >0.001, Hardy–Weinberg equilibrium (HWE), with *p*-value of 1 × 10^−6^, and variant calling genotyping coverage at ≥90%. In total, we examined >10 M variants. We additionally added sex, year of birth, and the initial 40 principal components (PCs), assessment centers, and sequencing batches, totaling of 172 covariates. For consistency, the same 172 covariates were included in the per-variant statistical regression protocol.

We used the summary statistics reported by Open Targets (OT) platform (release date: 9/2024; [38]) to collect the current knowledge on BC GWAS results. The BC used for the OT is a compilation from multiple resources including EFO: MONDO_0007254, UMLS: C0006142, NCIt: C9335. This broad definition includes the cases of Malignant Breast Phyllodes Tumor, Breast carcinoma, Breast lymphoma, Malignant breast melanoma and Breast sarcoma as described in OT. Each gene is listed with OT global score (range 0–1.0). Among these genes, 1825 genes are supported by any source of evidence, and 217 were associated with genetic-associated (GA) scores based on large-scale independent GWAS summary statistics [38]. Each locus is associated with genes by to their location and based on their association according to linkage disequilibrium (LD).

### 2.3. Gene-Level Effect Scores Across the Human Proteome

The PWAS framework [37] posits that causal coding variants affect phenotypes by altering the biochemical activity of their corresponding proteins. To quantify this, the pretrained ML model FIRM [37] estimates the impact of variants for each protein across the proteome. From ~97 million variants, 639,323 coding and splicing variants spanning 18,053 protein-coding genes were analyzed [34].

Each coding variant was assigned a functional effect score using FIRM predictor, a machine-learning random-forest-based classifier. FIRM is based on 1109 proteomic features that estimates the probability that a gene retains its protein function in the presence of that variant. It was trained against ClinVar pathogenic variants and demonstrated strong performance (AUC = 90%, accuracy = 82.7%). Variant effect scores are scaled from 0 (loss of function. LoF) to 1 (synonymous changes, no effect) [37]. PWAS then aggregated all variant-level FIRM scores to derive gene-level effect scores. These variant-level scores are aggregated within each individual to generate dominant and recessive gene-level measures, representing the probabilities of at least one or at least two damaging events in the gene. On average, each gene-level effect score reflects contributions from ~35.4 nonsense and missense variants. PWAS tests these gene scores for association with the phenotype using logistic regression under a combined model, in which a significant result indicates that one (or both) gene-level burden measures are associated with BC predisposition. For formal definitions and parameters used in FIRM and PWAS implementation see [37,39]. For these UKB analyses (for PWAS and GWAS), we included 172 covariates that covers sex, year of birth, 40 genetic principal components (PCs), genotyping batch (105 categories), and assessment center (25 categories), plus an intercept. We used the full set of 40 PCs to reduce potential false positives from population structure, accepting a slight reduction in statistical power (discussed in [39]). Based on testing the impact of UKB covariates on the logistic and linear regression, we showed that the genotype batch and assessment center can be safely removed as covariates [40].

To determine the effect size of a gene, we applied Cohen’s *d*. Cohen’s *d*, also known as the standardized mean difference, measures the difference between two means divided by the standard deviation (SD). In this study, Cohen’s *d* represents the normalized difference in mean gene effect scores between cases and controls (calculated independently for both dominant and recessive effect scores). Variant association and effect sizes from routine GWAS were computed using PLINK ver. 2.00a2.3LM with default logistic regression settings. We included a variance inflation factor (VIF) threshold of 5 to partially account for collinearity [41,42]. To more confidently assess the potential effects of multicollinearity and sensitivity, we computed VIFs (using statsmodels.stats.outliers_influence) for all 172 covariates. Almost all variants exhibited VIF values well below conventional thresholds, and only 0.3% (493 variants) had VIF > 50. Notably, none of them reached the multiple-testing-adjusted significance threshold. Thus, there is no indication that multicollinearity influenced inference.

The calculated z scores specify the effect size and its directionality. Note that in GWAS, a positive z score indicates a positive correlation between hypothyroidism and the number of alternative alleles, thereby indicating a risk variant. In PWAS, positive values indicate a positive correlation with the gene effect scores, whose higher values mean less functional damage. Thus, negative values are indicative of protective variants in GWAS versus risk genes in PWAS.

### 2.4. Transcriptome-Wide Association Study (TWAS) Analysis

We applied transcriptome-wide association studies (TWAS) [13], which integrate publicly available GWAS summary statistics with transcriptomic prediction models. Specifically, we used TWAS-Atlas (Ver. 1.0) built on European ancestry data. TWAS employs a framework which imputes gene expression across 44 human tissues simultaneously using a multi-task learning strategy [43]. Gene–disease associations is determined by combining association scores from single tissues into a cross-tissue test. TWAS offers a broad resource of models linking genomic loci to disease risk [13]. TWAS frequently yields an expanded list of candidate genes, as mapping of a locus to gene is often reports on >10 genes per locus. In this study, we did not examine coherence across different TWAS models.

### 2.5. External Comparative Analyses and Dependency Among Cohorts

To benchmark our findings, we compared them with results from gene-based genotype–phenotype associations available through ExPheWAS (https://exphewas.statgen.org/v1, accessed on 1 August 2025) [44]. ExPheWAS is a phenome-wide association study (PheWAS) platform that reports associations for 26,616 genes (including long non-coding RNAs), across a large number of phenotypes in ~410,000 UKB participants. We applied q-value < 5 × 10^−7^ as recommended for a gene-based analyses corrected for multiple phenotype testing. Additionally, we reviewed GWAS summary statistics from the Global Biobank Engine (GBE), which compiles data from >750,000 individuals across the UKB, the Million Veterans Program (MVP), and BioBank Japan. The phenotype studied is based on 16,136 cases of BC (indexed cancer1002) [45]. The GBE analysis applied consistent disease definitions and Bayesian methods to evaluate genetic effects. Notably, log10(Bayes Factor) ≥ 2 was considered to provide moderate evidence supporting the reliability of a gene or variant’s effect on a given phenotype [45].

Note that UKB, FG, and MVP are non-overlapping cohorts. PWAS uses UKB individual-level data after stringent filtering for kinship representatives and European ancestry. TWAS relies on UKB-based summary statistics from independent models, while ExPheWAS uses UKB with an independent gene-level model. OT aggregates and harmonizes external GWASs (from the GWAS Catalog and other sources). Thus, the BC studies partially overlap with MVP, but not with UKB or FG12. To mitigate the risk of spurious genetic signals arising from overlapping individuals, we avoid meta-analysis and instead employ cross-cohort replication to prevent artificially inflated associations.

### 2.6. Bioinformatics Tools

For functional enrichment of annotations and pathways, we applied the Gene2Func function of FUMA-GWAS (ver. 1.6, dates 6/2024) using default parameters and a set of genes as input [46]. All values are reported by their adjusted *p*-values, using the human gene coding proteome as background. For gene connectivity and protein–protein interaction (PPI) maps, we applied STRING (Ver 12.0, extraction date 6/2025) at a PPI connectivity score of ≥0.7 [47]. For knowledge graph, we applied the EnrichR-KB (https://maayanlab.cloud/enrichr-kg, accessed on 1 September 2025), a curated gene sets from many complementary sources like Gene Ontology (GO), KEGG, WikiPathway, DISEASE, and more [48]. The enrichment analysis is based on statistical methods considering multiple hypotheses. The report relies on the significant functional annotations where each finding is associated with a weighted combination of *p*-value and z-score (combined score).

### 2.7. Resource and Availability

The analyses shown in this study are supported by Supplementary Materials Figures S1–S5 and Supplementary Tables S1–S10. GWAS and PWAS performed in this study for ICD10: C50 (for female only, and both sexes). The results from these runs are shared in Zenodo data repository (10.5281/zenodo.17086606).

For summary statistics GWAS comparison we utilize the compilation from Open Targets (OT) platform https://platform.opentargets.org/disease/MONDO_0007254/associations (accessed on 1 June 2025). Exclusion and inclusion rules per outcomes and phenotypes from FinnGen are found in https://risteys.finngen.fi/endpoints/C3_BREAST_WIDE (accessed on 1 September 2025). GBE analysis used the phenotype names https://biobankengine.stanford.edu (index cancer1002, accessed on 1 June 2025). The UKB analysis from Neals lab summary statistics (2418 phenotypes) based on 337,159 s from ~337K unrelated white British individuals participants, resulted in 113 variants in https://pheweb.org/UKB-Neale/pheno/20001_1002#qq (accessed on 1 April 2025). Lavaa plot visualization application generated genetic volcano plots were available in PheWAS browser lined to FinnGen https://mvp-ukbb.finngen.fi/pheno/C3_BREAST_EXALLC leading to selected variants in https://mvp-ukbb.finngen.fi/variant (accessed on 1 August 2025). ExPhWAS browser is available in https://exphewas.ca/v1/docs/browser (accessed on 1 August 2025) and allows searching for the breast cancer phenotype of Phecode 174.11 (female only).

## 3. Results

### 3.1. Integrative Framework for Breast Cancer (BC) Gene Identification and Validation

The goal of this study is to compile a set of genes that can be confidently assigned as a predisposition BC core set. The genetics of BC have been extensively studied. However, results from complementary association studies and gene-based methods remain inconsistent. As a result, a gap persists between BC genetics, risk assessment, and clinical application. Here, we present a framework that seeks coherence across large population studies, validates BC core genes, and proposes clinical utility.

**Figure 1:**
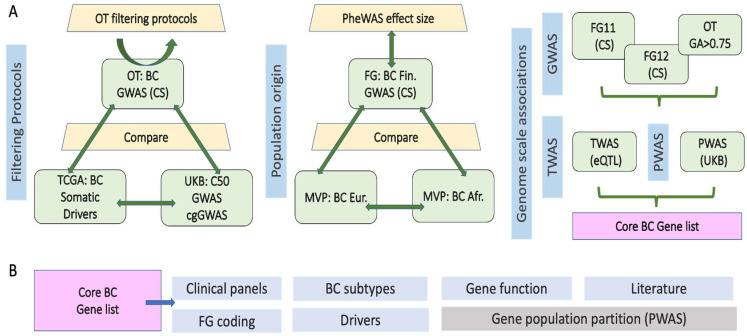
The outline of the framework presenting for BC core gene set and validation. (**A**) The protocol used for filtering the OT gene list based on multiple BC GWAS. Testing GWAS genes with BC driver genes from somatic data. The diversity of the population origin in illustrated by MVP data from European and African origin. A complementary set of GWAS, PWAS, and TWAS was combined to present a core BC gene list. (**B**) Validation through external resources, the utility of population partition is tested for PWAS results. BC, breast cancer; FG, FinnGen; OT, Open targets; UKB, UK biobank; CS, credible variant set mapped to genes.

Figure 1A illustrates the multi-layered approach used in this study to identify and validate core BC genes. The main strategies include several stages (Figure 1A, left to right). These include firstly applying filtering protocols using the Open Targets (OT) platform based on results from multiple GWAS and their credible sets (CS), and then comparing germline results to somatic BC driver gene lists to test the contribution of established cancer genes to BC predisposition. We further leveraged classical and coding-gene restricted GWAS (cg-GWAS) from the UKB cohort to assess gene-level coherence and replication in an independent cohort of FinnGen (FG). For estimating population–origin coherence and variant effect sizes, the Finnish GWAS results (FG12) with summary statistics from the Million Veteran Program (MVP) were partitioned to include European- and African-ancestry BC cohorts. Lastly, we integrated complementary protocols from OT-filtered genes identified through GWAS, and other genome-wide association methods such as TWAS (based on eQTL) and PWAS (based on predicted protein function impact). Figure 1B presents the converged results from these approaches, yielding the core BC gene list. These genes are further validated using external evidence, including clinical panels, FG coding, BC subtype data, somatic drivers, gene function, and published literature. Finally, this framework demonstrates the clinical utility of evaluating population stratification in relation to BC risk.

### 3.2. Germline Risk Genes for BC and Somatic Driver Genes Are Largely Distinct

**Figure 2:**
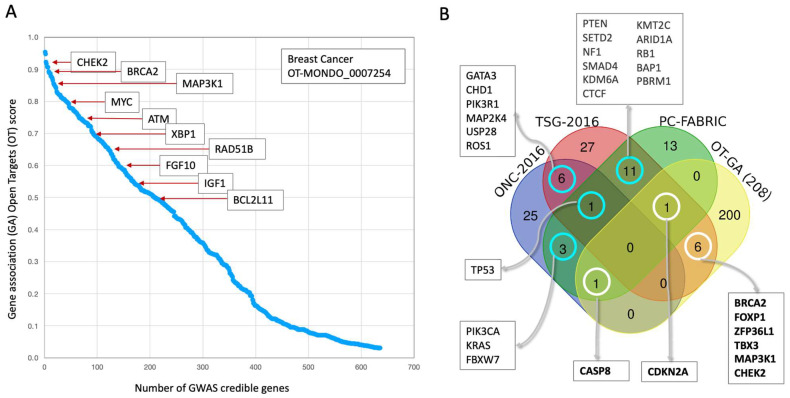
GWAS BC-credible genes from OT platform and overlap with driver genes. (**A**) A plot showing the distribution of genetic association (GA) scores for 638 genes identified by the OT platform, with a few selected genes that have a high GA score ≥ 0.5 (total 208 genes). (**B**) A Venn diagram illustrating the overlap between three gene sets. A list of 208 BC-credible genes from the OT platform (with GA ≥ 0.5), a list of driver genes from primary BC samples, partitioned to oncogenes (ONC, 36 genes) and tumor suppressor genes (52 genes, TSG) [32], and a pan-cancer 30 driver genes from FABRIC [49]. The diagram highlights the minimal overlap, with 8 genes from the OT list also identified. The diagram also shows the overlap between the FABRIC list and the ONC and TSG lists from primary BC samples, noting that 6 genes appear in both the ONC and TSG categories, indicating their context-dependent function.

We investigated the relationship between germline risk genes and somatic driver genes for BC. GWAS results for BC were compiled using the OT platform, which assigns a genetic association (GA) score to each gene (Figure 2A). Out of 638 genes with a GA score, we focused on the subset with GA score ≥ 0.5. As anticipated, *CHEK2* and *BRCA2* were ranked at the top. However, approximately 100 genes also demonstrated high GA scores (>0.7), suggesting a broader genetic landscape for BC predisposition than previously assumed (Supplemental Table S1).

To determine if BC predisposition genes share a function with cancer drivers, we compared the list of the top 208 OT genes with a preselected GA score ≥ 0.5, with established BC-derived driver gene lists. We used two primary resources for driver genes: (i) a comprehensive analysis of 2433 BC samples that identified oncogenes (ONC) and tumor suppressor genes (TSG) [32]. (ii) FABRIC, a machine learning method developed to detect pan-cancer drivers from TCGA data [36]. FABRIC identifies cancer genes under positive selection by comparing the impact of variants on protein function against an expected baseline [49].

Our analysis showed minimal overlap between the GWAS-based predisposition genes from the OT platform and the somatic driver gene lists (Figure 2B). Although there was a statistically significant overlap with the TSG list (hypergeometric *p*-value < 0.01), there was no enrichment of driver genes among the GWAS derived BC predisposition genes. Our findings confirm that only a small subset of drivers is detectable from large-scale cohorts, with 200 of the 208 genes showing no evidence of being cancer driver genes. The overlapping with TSGs included *BRCA2*, *FOXP1*, *ZFP36L1*, *TBX3*, *MAP3K1*, and *CHEK2*. Additionally, *CASP8* and *CDKN2A* were identified through their overlap with FABRIC and assigned as ONC and TSG gene, respectively. Notably, a polymorphism in *CASP8* (rs1045485) has been shown to significantly reduce BC risk in certain European and Chinese populations [50,51]. *CDKN2A* is a key gene in the cell cycle which was initially associated with melanoma-prone individuals. However, it was shown to increase the prevalence of a broad spectrum of cancer types [52].

We concluded that while GWAS-identified gene lists are likely to be noisy due to false discovery, the small set of genes with germline mutations that also function as somatic BC drivers, enriched by TSGs, are strong candidates for BC predisposition.

### 3.3. Prioritizing Candidate Genes from the Abundance of GWAS False Positive Genes

To prioritize the list of potential BC susceptibility genes, we focused on 208 candidates with GA score ≥ 0.5. We then looked for genes within this subset that had a higher global score relative to their GA score reported by the OT platform. This approach allowed us to select genes supported by independent evidence, such as data from scientific literature, animal models, burden test, ClinVar and genetic constraints. Using this protocol, we identified 10 genes (Table 1). Only a few of the genes, like *ATM*, *BRCA2*, and *RAD51B*, were already well-known BC predisposition genes, while others were not. Some had been reported as driver candidates in somatic BC samples but were not considered germline BC predisposition genes. Comparing the OT global score relative to GA score per gene, demonstrates that prioritizing genes from GWAS can be used for removing false positive and ranking genes based on the strength of independent, external evidence.

To further improve our discovery rate, we inspected the subset of 208 genes with high GA scores (≥0.5) relative to the global score. We analyzed the set of gene by their functional enrichment test using FUMA-GWAS. From an initial list of 26 genes (Supplementary Table S1), we identified eight that were significantly enriched in at least two of the four tested gene sets: *PEX14*, *WDR43*, *CCDC170*, *DNAJC1*, *ZNF365*, *CCDC88C*, *TOX3*, and *FTO* (Table 2). Out of all GWAS experiments reported by FUMA-GWAS, all results (at *p*-value < 1 × 10^−4^) resulted in a strong enrichment for phenotypes related to BC and breast physiology.

Manual inspection of genes with a GA score > global OT score, coupled with solid statistics across multiple GWAS (Table 2) confirmed the relevance of several BC predisposition candidates. *PEX14*, a gene involved in peroxisome biogenesis, was recently implicated in BC predisposition by a family-based study [53]. *CCDC170*, which is located adjacent to the ESR1 gene, shows a consistent association with triple-negative BC (TNBC) across multiple GWAS. Fusion events and specific risk alleles have been linked to aggressive BC subtypes, with a particularly strong effect in the East Asian population [54]. Variants in *ZNF365* have been repeatedly associated with BC risk, especially in individuals with a *BRCA2* mutation [55]. *TOX3* is a known low-penetrance BC susceptibility gene, with common risk variants found in both European and Asian cohorts. Lastly, *WDR43* was validated as an ER-negative susceptibility locus through meta-analysis and further confirmed by functional and eQTL studies [56].

We confirmed that evidence-based consideration is valuable for validating known and new candidates as BC predisposition genes (Table 1). We also proposed that despite a lack of strong non-genetic evidence, valid candidates display a consistent enrichment in BC-related phenotypes from independent GWAS findings (Table 2). Although further confirmation is needed, this simple ranking protocol improves the discovery rate while removing a large fraction of false positives.

### 3.4. Germline Risk Genetics for BC Is Sensitive to Population Origin

We performed routine GWAS on the imputed variants from UKB. We have used BC diagnosis (ICD-10 C50) and included 10 covariates (sex, age, and top 8 strongest PCs to account for major population structure). There are 16,952 cases and 409,651 controls (among them, 216,254 females). There are only 139 BC cases among the male cohort. We performed the GWAS analysis for both sexes and identified 2727 variants that met the whole genome GWAS threshold of *p*-value < 5 × 10^−8^. Among them, only 31% of the variants are associated with a gene (i.e., resulted from a successful variant-to-gene mapping). The rest of the GWAS-identified variants are intergenic, and will be further discussed. Altogether, we report on 137 different genes (Supplementary Table S2). Based on the assumption that among the GWAS there are many false positives, we filtered out the list by the following criteria: (i) We only considered variants with minor allele frequency (MAF) of >1% to avoid statistical biases. This step already removed 15% of the significant variants. (ii) We concentrate on genes that are supported by at least two variants with the vast majority of the variants of a gene shares the same directionality. A filter for multiple occurrences of variants for the same gene further confirms the gene relevance, its effect size (measured by the Odds ratio, OR) and coherence in directionality. There are 32 such genes. The other 105 genes were supported by a single statistically significant variant and therefore were excluded. Notably, extreme cases were detected with many variants per gene as for *NEK10* (264 variants), *FTO* (82 variants), *TOX3* (67 variants), *EGFR2* (59 variants), *MAP3K1* (50 variants), and *MRTFA* (45 variants). Not only were these genes validated, but their effect size was substantial. For example, the OR values of *EGFR2* and *TOX3* are approximately 1.28 and 1.24, respectively.

**Figure 3:**
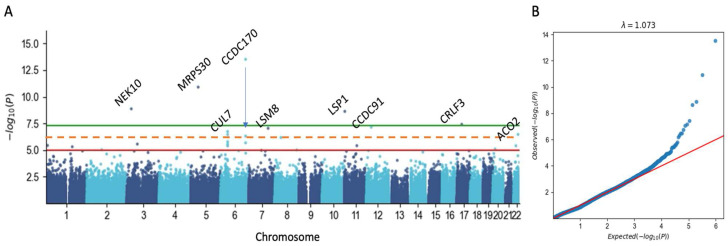
The GWAS results for predisposition in BC. (**A**) Manhattan plots for ICD-10 C50. The plots show the significance of all the variants and genes tested with cgGWAS (see Methods). Among the 488,783 variants in the female cohort, only 11 variants were mapped with an exome-scale threshold of 5 × 10^−7^ (Supplementary Table S3, listed all variants with *p*-value < 5 × 10^−6^), with two genes represented by two significant variants (*CUL7* and *CCDC170*). The gene names are indicated by the genes meeting the dashed red line threshold at −log10(P) of 6.3 representing *p*-value < 5 × 10^−7^. The red and green lines indicate a threshold of 1 × 10^−5^ and 5 × 10^−8^, respectively. Note that ChrX and ChrY were not included in the analysis. (**B**) QQ plot comparing the distribution of observed and expected distribution log of the calculated *p*-values of the null hypothesis (i.e., no associations, red line). Inflation of test statistics (γ) confirms the minimal inflation.

To enhance the interpretability, we performed restricted GWAS while limiting the variants to be located in gene length of coding genes (called cgGWAS). There are 531,246 variants that met the coordinated of coding genes (18,053 genes and the included imputed variants), among them 488,783 variants were mapped in the female cohort. Only 11 variants complied with an exome-scale threshold of 5 × 10^−7^ with two genes that were represented with two variants each (*CUL7* and *CCDC170*; Supplementary Table S3). Figure 3A shows the Manhattan plot for the female cohort for IDC-10 C50, indicating the 9 significant genes. The vast majority of the statistically significant variants (>95%) are rare and ultra-rare (AF < 0.001). The variants associated with *CUL7*, *LSM8*, and *ACO2* (Figure 3A) are based on ultrarare variants (AF < 0.001). Figure 3B shows the quantile-quantile (QQ) plot that compares the distribution of observed and expected distribution of the *p*-values under the null hypothesis (i.e., no associations). The systematic inflation of test statistics (γ = 1.073) confirms the insignificant inflation across all datapoints. While the GWAS included covariates, we concluded that the GWAS results are not stable enough to provide a solid and reliable BC predisposition gene list.

We then tested how using different populations can provide insight into the genetic mechanisms driving BC risk. We used a cohort from FG12 dataset, which defines BC cases as malignant neoplasm of breast while excluding all other cancer occurrences from the control group (Supplemental Figure S2). This compilation included 24,978 participants (abbreviated C3_BREAST_EXALLC).

**Figure 4:**
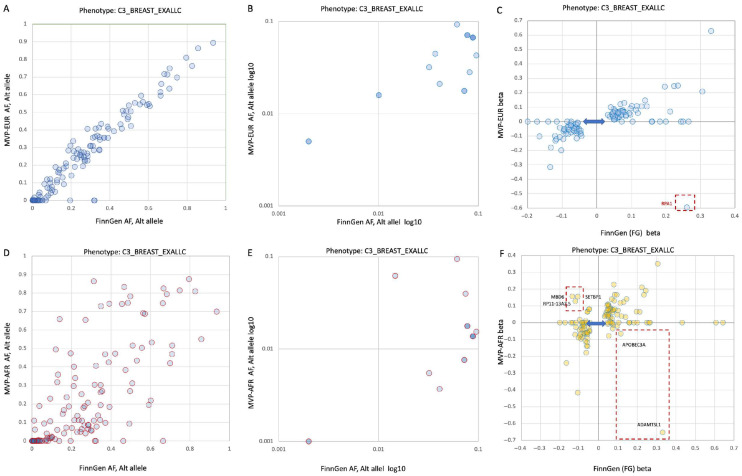
Comparisons of BC-associated variants from B3_BREAST_EXALLC meta-analysis between the FinnGen (FG) cohort and the MVP cohorts. (**A**) Scatter plot of the allele frequencies (AF) between individuals in FG12 and MVP participants of European ancestry (MVP-EUR). (**B**) Scatter plot of the AF between individuals in FG and MVP participants of European ancestry (MVP-EUR) for the AF range of 10% to 0.1% in the alternative allele (log scale). (**C**) Compares the effect sizes (beta, β) of these same BC-associated variants between the FG and MVP-EUR cohorts. The red dashed box highlights a variant with a significant difference in effect size between the two populations. Double-sided arrows indicate variants present in the FG cohort but missing from the MVP-EUR cohort. (**D**) Scatter plot of the AF between individuals in FG12 and MVP participants of African ancestry (MVP-AFR). (**E**) Scatter plot of the AF between individuals in FG12 and MVP-AFR of 10% to 0.1% in the alternative allele (log scale). (**F**) Compares the effect sizes (β) of variants between the FG12 and MVP-AFR cohorts. The red dashed boxes highlight variants with notable differences in effect size, including some that show an increased risk in the FG cohort but a protective effect in the MVP-AFR cohort (e.g., *ADAMTSL1*, *APOBEC3A*). Conversely, variants in *MBD6*, *SETBP1*, and *RP11-13A2.5* show an opposite trend. This suggests potential ancestry-specific effects or differences in linkage disequilibrium (LD). A full list of all 137 associated variants and their statistical information is available in Supplementary Table S4.

We then analyzed the coherence of associated variants by comparing the FG12 cohort to the Million Veteran Program (MVP), which was split into European and African ancestry groups, as well as the UKB cohort which does not overlap with FG. Figure 4 compares the allele frequencies (AF) and effect sizes of 137 variants identified in the FG12 meta-analysis cohort to those in the MVP European and African groups. A notable finding was that 26% of the variants from FG12 were missing from the MVP’s European-ancestry group. This is consistent with the importance of founder effects and population bottlenecks in the Finnish population, and the noisy GWAS results.

Figure 4A shows that the variant frequencies between the Finnish and other European populations are generally similar, as most data points are close to the diagonal line. This is further supported by Figure 4B, which shows the same trend for rare variants. Figure 4C shows a good agreement in the effect sizes (β) between the FG12 and MVP European-ancestry (MVP-EUR) cohorts. However, about a quarter of the variants from the FG cohort did not have a match in the MVP-EUR data, which is likely due to founder effects and population bottlenecks specific to Finland [22]. A similar analysis was performed for the MVP African-ancestry (MVP-AFR) group (Figure 4D). Here, 29% of the variants from the FG cohort could not be matched. The data points for this comparison are more scattered, indicating a significant difference in allele frequencies (AF) between the two populations. This trend is also evident for less common variants (Figure 4E). Figure 4F displays the effect sizes (β) for the FG12 and MVP-AFR cohorts. While there is a general agreement, some variants showed marked discordance in their effect size, with some even having opposite effects (e.g., increasing risk in one population but being protective in the other). These variants, associated with genes like *ADAMTSL1*, *APOBEC3A*, *MBD6*, *SETBP1,* and *RP11-13A2.5*, suggest differences in LD or ancestry-specific effects. We also considered the possibility that some missing variants were due to technical or methodological inconsistencies in quality criteria, genotyping of the DNA array and imputation protocols.

We hypothesized that genes with a strong effect size (β > |0.1|) carry a stronger impact on population-specific risk variants. For instance, a missense variant in the *RPA1* gene showed a strong effect in the FG12 population (β = 0.261), but a conflicting association in the MVP-EUR group (Figure 4C). *RPA1* is involved in DNA repair and is functionally connected to the *BRCA1/2* pathway. We confirmed that this apparent conflict was due to a small number of cases in the MVP cohort, meaning that in this case, the conflicting trend was not statistically significant.

### 3.5. A Pleotropic Nature of BC Associated Variants with Moderate Effect Size

**Figure 5:**
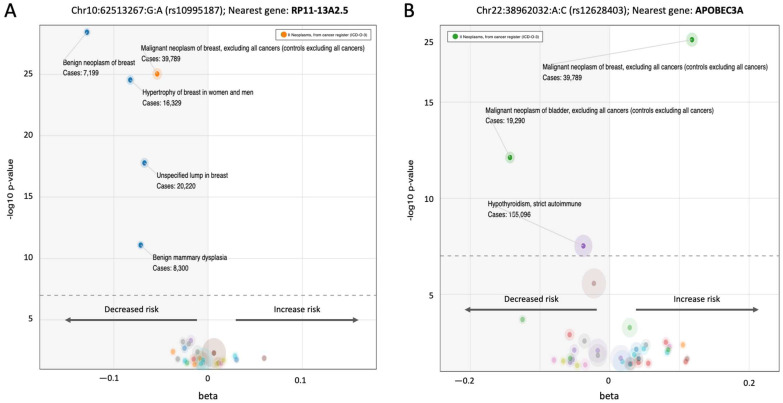
Lavaa plot representation from PheWAS for FG associated genes (FG12) with moderate effect size (b > |0.1|). Each plot visualizes a single nucleotide variant (SNV) the effect size (b, x-axis) and its direction and the significance of the association by log_10_(*p*-value). The dashed horizontal line indicates the genome-wide significance threshold (*p*-value 5 × 10^−8^). Negative beta values (left side, gray background) indicate a protective effect, or a decrease in risk. Significant phenotypes are listed along with the number of cases. Only significant phenotypes are annotated. The size of each bubble corresponds to the number of cases for that specific trait. (**A**) Chr10:123,232,597-A (rs10995187). This plot shows associations for a variant near the RP11-13A2.5 gene. (**B**) Traits association to chr22:39,599,242-C. This plot shows associations for a variant near the *APOBEC3A* gene. Color indicate different classed of PheWAS phenotypes. A full list of the 137 significant variants, including their ethnic distribution and associations in other cohorts, is provided in Supplemental Table S4.

The existence of common BC predisposition variants across multiple populations may be explained by their pleiotropic effects [57]. Comparing the FG12 cohort with the MVP African-ancestry (MVP-AFR) cohort revealed several variants with inconsistent effect sizes, sometimes with opposing directions (Figure 4F). Figure 5 illustrates the pleiotropic nature of these variants, showing their representation in PheWAS and their ancestry-specific effects (Supplementary Table S4). Of the 137 BC-associated loci identified in the FG meta-analysis, 42% had a moderate effect size (β > |0.1|). Figure 5A analyzes a specific variant, rs10995187, located next to RP11-13A2.5, which is a lncRNA gene with an unknown function. This variant has an AF of 20% in Europeans but is extremely rare in East Asian populations (AF: 1.3 × 10^−3^). It shows a significant negative association with both malignant BC and benign breast conditions, suggesting it may play a protective role in various aspects of breast physiology.

The case of the *APOBEC3A* gene-associated variant further highlights this complex nature, as it shows a strong protective effect against bladder cancer but a significant increased risk for BC. The coexistence of these conflicting trends, with positive and negative beta values demonstrates that variants can have a specific, and sometimes opposing, impact on different cancer types.

The pleiotropic nature of a variant adjacent to the *TTC28* gene (Chr22:28365160:C:T, rs62237617) is illustrated in Supplementary Figure S3. The *TTC28* gene is involved in centrosome regulation and genomic stability. This single variant is significantly associated with 10 different traits, many of which are malignancies (e.g., breast, thyroid, colon, bladder, and kidney cancers) but also include conditions related to genome stability in blood cells, such as leukemia and lymphoma. The strong pleiotropic effect of a variant affecting *TTC28* gene highlights it as a potential, previously overlooked, predisposition risk gene for multiple cancer types. We conclude that examining PheWAS for pleiotropic effects is a valuable method for identifying variants according to their potential impact on many traits. It also shows that BC predisposition may impact non-coding genes (Figure 5A), while variants may also exhibit opposing risk directions (Figure 5B). We argue that pleiotropic variants contribute to the understanding of biological pathways, thus improving interpretability for risk prediction. Carriers of pleotropic variants in the *TTC28* gene may face elevated risk for multiple cancers.

### 3.6. Gene-Based Functional Model of PWAS with Inheritance Mode

The PWAS is a gene-based model that aggregates the effects of each variant within the gene to assess the combined effect of damage to the protein function at an individual. The difference in the values of the gene-effect size for cases and controls determines the PWAS statistics [39]. For the group of European patients diagnosed with BC (ICD-10 G50; covering 10,682 and 138,504 female cases and controls, respectively), PWAS identified two genes based on variants affecting the coding (and splicing sites within) in females. Both genes *CHEK2* and *CCDC170* were identified with a dominant inheritance mode (Supplementary Table S5) [34,39]. Note that when all Europeans were included (both sexes) also *TSC22D3* (ChrX, also called *GILZ*) was identified as significant. ICD-10 C50 is identical to the participants included in Phecode PP174.11 (Malignant neoplasm of female breast; Supplemental Figure S1).

As shown (Figure 4), ethnic groups beyond Europeans increase diversity. We therefore conducted PWAS test on the global UKB and used the UKB recent version (version Q3, 2022) to update the number of patients diagnozed by ICD C50. Supplementary Table S5 shows the gene-based significant list for genes from females diagnosed with C50 (16,839 cases; 216,529 controls), identifying eight genes (*NEK10, CCDC170, CHEK2, MRPS30, MTMR11, CRLF3, LSP1*, and *FKBP5*). While the PWAS method provides statistics for each gene on the assumption of a dominant, recessive or hybrid mode of inheritance [34], none of the eight identified gene is supported by a recessive inheritance. However, with a unified number of UKB cases (16,975) within the entire population (males and females, with 410,114 controls), more genes were identified (total 12). Among these additional four genes, two genes are located in ChrX (*TSC22D3* and *CHST7*), with *CHST7* and *C4BPB* being supported entirely by a recessive inheritance. The inheritance mode associated with any of the 12 PWAS identified genes is illustrated in Supplementary Figure S4.

We confirmed these findings by testing the results from the FG12 dataset. Note that the FG analysis is not restricted to the coding region. We found that two of the genes were also identified as BC predisposition genes in FG12. *CHEK2*, a BC predisposition gene, was supported by three variants, the most significant being an intron variant (rs186430430, AF 0.8%) with an odds ratio (OR) of 2.42 (*p*-value 2.6 × 10^−78^). *CCDC170* was identified through an intergenic variant (rs6900157, AF 21.6%) with an OR of 1.1 (*p*-value 5.0 × 10^−16^). Notably, *CCDC170* was also found on the OT platform as a gene that remained after filtering for false positives (Table 2). However, the gene *TSC22D3* was not detected in the FG12 dataset. We conclude that the very conservative gene list of PWAS reduces false positive discovery (discussed in [39]). Moreover, it presents the value of considering gene recessive inheritance, where at least two damaging mutations affect both alleles of a gene per an individual.

### 3.7. Robust BC Predisposition Gene List and Transcriptome-Based Associations

We aimed to create a robust catalog of BC susceptibility genes by integrating and confirming results across different populations and study types. Instead of simply using the joint meta-analysis results from FG12, UKB, and MVP, which together included 39,789 cases and 1,002,960 controls, we focused on the credible gene list from FG12.

**Figure 6:**
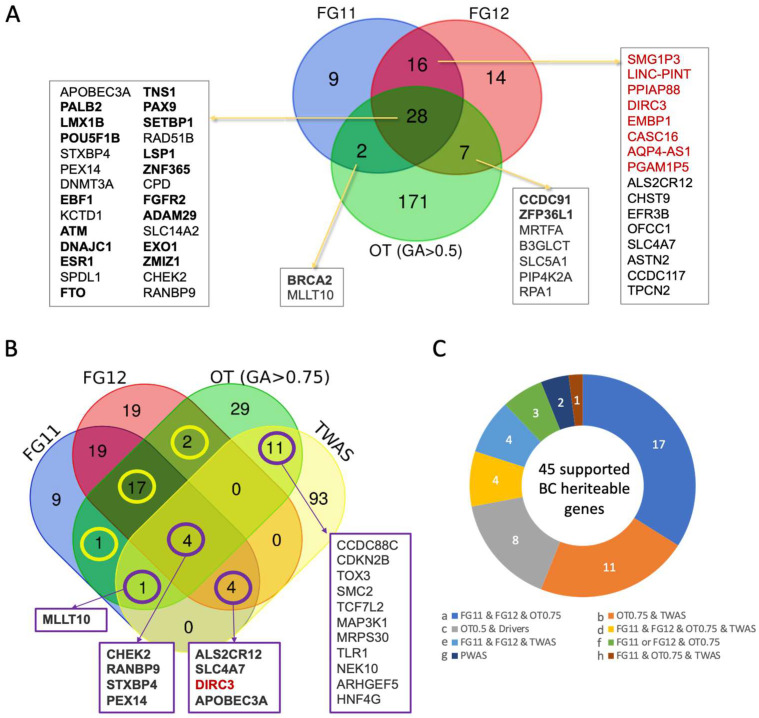
Venn diagrams of gene lists across cohorts. (**A**) FinnGen credible variants and the associated genes from freeze 11 (FG11), freeze 12 (FG12), and the list of 208 genes from the OT at GA score > 0.5. Detailed summary statistics of the credible variants set (CS) for FG11 and FG12 are available in Supplementary Tables S6 and S7, respectively. Shared genes are listed, and in bold are the genes that are compiled with a stringent GA score > 0.75 that account for the top 3% of the genes with genetic association evidence (20 genes). Coding genes are shown in black and lncRNAs that are shared between FG11 and FG12 are in marked red. (**B**) Venn diagram of FG11, FG12, OT with GA score > 0.75 (OT0.75) and genes associated with 79 TWAS loci. TWAS details statistics are provided in Supplementary Table S8. The yellow circles are the 20 genes as listed in A, and the purple circles are genes shared with TWAS results. In bold faces are genes with strong evidence and are included in the core BC predisposition genes. Red font indicates lncRNAs. (**C**) Pie chart of all the core BC predisposition genes by their evidence (labeled a–h). The data are based on the Venn diagram in A and B. OT at GA score > 0.5 and >0.75 are labeled OT0.5 and OT0.75, respectively. The list of 50 genes with evidence represents 45 unique genes.

To build a consensus catalog, we first checked for consistency within the same population. We compared FG freeze 11 (FG11; 20,586 cases and 201,494 controls) to freeze 12 (FG12; 24,270 cases and 222,078 controls). The overlap between the gene lists from FG11 and FG12 was highly significant, with a majority (62%) also overlapping with the OT list of genes with GA score ≥ 0.5. This overlap was extremely significant (hypergeometric *p*-value 2.8 × 10^−21^) (Figure 6A). We identified a core set of 20 highly confident BC predisposition genes, including 17 that were shared among all three lists and 3 additional genes from FG11 or FG12 that had strong OT support (GA ≥ 0.75). Note that while FG data also cover non-coding RNAs, the OT list, only consider coding genes.

To expand this 20-gene core list, we applied transcriptome-wide association study (TWAS) method for gene discovery. This method links genetic variants to disease by predicting gene expression from genetic data and then testing if this predicted expression is associated with the trait. TWAS is particularly useful for identifying variants in regulatory elements, which can have a strong effect on gene expression but may not be picked up by the traditional association methods.

We identified 79 statistically significant loci in TWAS analysis (Supplementary Table S8). TWAS mapping from variant to gene is even more challenging that in classical GWAS. A single locus can be associated with multiple genes (2 to 10), making a precise locus-to-gene (L2G) assignment difficult. The resulting TWAS significant finding of 79 loci (Supplementary Table S8) is expanded to 141 listed genes (125 unique) among them 13% are lncRNAs (16 genes). We found 20 genes from the TWAS analysis that were also supported by at least one other gene set (Figure 6B). The most confident group of shared genes, found in TWAS, both FG cohorts, and the high-confidence OT set, included *CHEK2*, *RANBP9*, *STXBP4*, and *PEX14*. While *CHEK2* is a known predisposition gene, the others were not previously directly associated with BC predisposition.

Table 3 lists the genes supported by confident evidence from our analysis. This list compiles results from complementary approaches to BC genetics, including GWAS, PWAS, and TWAS, together with an assessment of evidence quality. Based on these sources (labels a–h), 45 unique genes were identified. Evidence from FG12 coding variants (*p*-value < 5 × 10^−7^) further reinforced our findings. Although some associated variants had AF < 0.5%, rare variants were not explicitly included. Table 3 shows that 15 of 30 reports of highly significant coding variants within genes listed in FG12 are included in the core BC susceptibility gene list. Of these, 11 genes have the literature support for BC predisposition (binomial test, *p*-value 1.47 × 10^−3^). This confirms that previous knowledge captured the importance of coding variants within genes. To substantiate the evidence and support for the core BC list (total 38 genes), we included as evidence the ExPheWAS significant list (q-value < 5 × 10^−7^, Supplementary Table S9) based on 17,314 cases and 192,986 female controls from UKB. The list from ExPheWAS includes 21 genes, 9 are non-coding belong to lncRNAs, and another 9 support the findings in Table 3. Several genes are supported by multiple variants, and significant evidence was linked to multiple BC-related phenotypes (Table 3). For example, *CCDC170*, identified by PWAS (evidence g; Supplementary Table S3), is additionally supported by FG with two variants and six breast-related phenotypes (Table 3). Note that 11 genes are supported only by evidence b (OT0.75 and TWAS, Figure 6C) are not validated (marked with an asterisk and gray background in Table 3) and 7 such genes lacking any further support are to be excluded from the high confidence gene list. Following filtration, the BC predisposition includes 38 core genes. Notably, for about one-third of these 38 genes, there is no prior literature linking them to BC risk. Interestingly, several genes that signify subtypes of BC (e.g., ER positive or ER negative, Supplementary Table S10) are included in the core set of 38 predisposition genes (Table 3, Supplementary Figure S5). We tested the current presence of clinical panels and marked 4 of the genes that are already included in commonly used Invitae and Myriad MyRisk hereditary BC panels (Table 3). Inspecting the functional annotation of the BC predisposition core set emphasize the importance of genes acting in DNA repair, cell cycle, adhesion, hormonal regulation, epigenetics, and transcription regulation.

### 3.8. Core BC Predisposition Genes Are Highlight Processes of DNA Integrity and Stability

**Figure 7:**
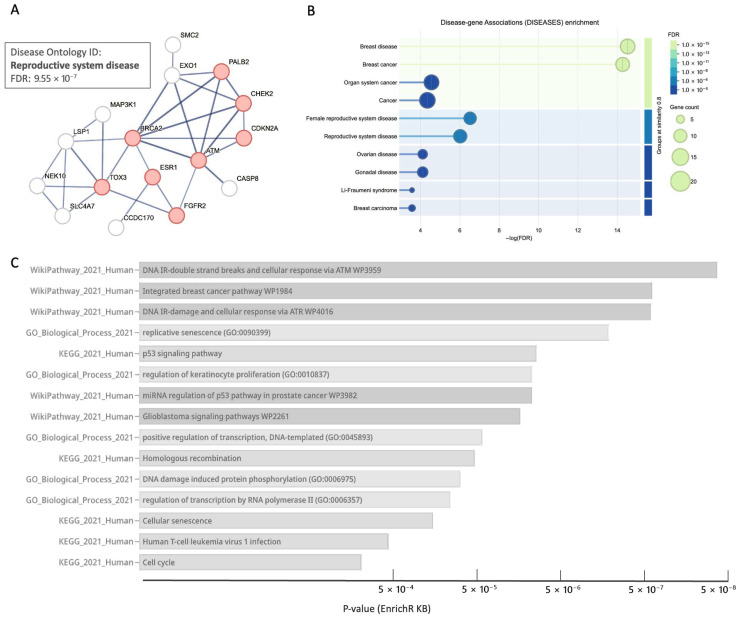
Functional view of BC predisposition genes. (**A**) The 38 core BC genes were analyzed by STRING PPI network (at PPI confidence score ≥ 0.7). The connected network is very significant (PPI < 1 × 10^−16^). The genes in red nodes are assigned with annotation from disease ontology (DOID of reproductive system diseases). (**B**) Enrichment of the core BC gene by STRING according to annotation of DISEASES. Note the significance of genes that are shared with other gynecological diseases (q-value FDR). (**C**) Bar view from the EnrichR KB analysis according to the *p*-value of the enrichment based on a combination of the databases of human DISEASES, KEGG, Gene ontology (GO) for biological process, and WikiPathways. All 15 listed annotations are significant.

To assess the functional relevance of BC predisposition genes, we performed enrichment analysis on the 38 core BC genes. Most genes were connected in a single, highly significant protein–protein interaction (PPI) network (STRING, confidence ≥ 0.7, PPI *p*-value < 1 × 10^−16^; Figure 7A). Cross-mapping these genes against the DISEASES database revealed significant enrichment for other reproductive-system diseases, suggesting a shared etiology (Figure 7B, FDR-corrected q-values). An integrative EnrichR query combining human DISEASES, KEGG, gene ontology (GO) biological processes, and WikiPathways identified 15 significantly enriched pathways and processes (Figure 7C), highlighting a role for DNA damage repair, cell-cycle checkpoints, transcriptional regulation, cellular senescence, and maintenance of DNA integrity.

These results indicate that the primary functional modules through which inherited variation drives BC risk are tightly connected to DNA recombination, repair, and cell-cycle control. Similar enriched annotations extend to other gynecological diseases, including ovarian cancer and broader reproductive disorders.

Importantly, gene-based partitioning of populations, largely overlooked in classical GWAS, has clinical value. Screening for BC-core genes may benefit high-risk families and populations and inform targeted therapeutic strategies by focusing on cellular pathways commonly affected across these genes.

## 4. Discussion

This study revisits current knowledge on BC predisposition genes and proposes a protocol for ranking strongly supported susceptibility genes while minimizing false positives and weakly supported candidates. A major source of false positives stems from the uncertain mapping of lead SNPs to genes. In gene-dense loci, linkage disequilibrium (LD) can link dozens of genes to a single associated variant [58]. Here, we focused on the gene level rather than on individual leading variants. Traditional GWAS typically assign an association to the nearest gene, an oversimplification that overlooks complex genomic architecture. Even when chromatin interactions are incorporated, regions with poorly annotated genomic structures often suffer from incorrect gene assignments [59,60]. Although GWAS has uncovered hundreds of common risk loci for BC, these variants individually confer modest risk and collectively explain only a limited fraction of heritability [61]. Despite advances in fine mapping and co-localization approaches [62], much of the inherited risk remains unexplained. For example, a systematic analysis of conservative BC genes identified 15 new loci explaining only ~2% of familial risk [30], while other estimates suggest that low-penetrance loci together account for less than 4% of familial relative risk [18]. In total, over 80 known loci explain roughly 16–18% of familial risk [30,63]. In this work, we integrate complementary approaches from GWAS, TWAS, and PWAS, emphasizing coherence across data sources rather than merging them into a single cohort. While requesting coherence may reduce statistical power, it enhances consistency, mitigating artifacts arising from founder effects or technical limitations in variant mapping and imputation [64,65].

In this study, we have considered rich collection of 172 covariates that were included in the GWAS and PWAS methods [40,66]. However, future research could benefit from advanced correlation and information-theoretic measures that capture both linear and nonlinear dependencies in physiological signals [67]. Incorporating these methods may provide a more comprehensive understanding of underlying signal interactions [68]. By maintaining a gene-centric perspective, we argue that the conservative framework adopted here enables more robust mechanistic and functional interpretation. The foundation of PWAS is the impact of mutations on the function of the encoded protein. Therefore, it is resistant to mapping issues that strongly affect other association methods (e.g., TWAS and GWAS). Notably, including changes in gene expression data as a proxy for genetic signal through TWAS neither contributed to nor altered the risk associations of candidate genes, consistent with observations from a large study of more than 46,000 women with BC [69]. Instead, PWAS is based on a very conservative approach with a small number of gene discoveries [34]. Nevertheless, the listed genes often lead to functional interpretation of biological mechanisms. This helps identify causal genes and functional pathways more accurately. Another advantage of gene-based approaches is the ability to capture compound heterozygosity and recessive inheritance patterns, which are typically missed by GWAS. Notably, all PWAS results were further supported by other evidence (Table 3), confirming them as high confidence. Of the PWAS-identified genes, recessive inheritance is exposed. Recent PWAS findings suggest that compound heterozygosity at multiple DNA repair genes plays a larger role in BC predisposition than previously recognized [39].

Another factor that can weaken genetic signals is the inconsistency in the definition of the BC phenotype. Statistical outcomes are sensitive to cohort composition; in the case of BC, it also matters if males are included in the cohort, differences in average age across biobanks, and if the accuracy of clinical records is not always standardized. In the FG dataset used throughout this study, BC patients afflicted with other cancer types were excluded, whereas for the TWAS analyses, self-reported BC data were used. Consequently, our core BC predisposition gene set (Table 3) does not include broadly tumor-associated genes such as *TP53*, *PTEN*, *CDH1*, and *STK11*, which have been implicated across multiple cancer types and are confirmed to contribute to BC predisposition risk [70]. Because we seek overlap between methods and resources, and used FG cohorts that were depleted of other cancers (Figure 6, Table 3), some classical predisposition genes that play a central role in pan-cancer predisposition were therefore excluded (e.g., *TP53*, *PTEN*) [71].

Expanding the catalog of BC predisposition genes according to clinical panels is critical for genetic counseling, follow-up, and targeted family screening [31]. In this study, we focus on BC cases in a broad case–control framework. We ignore the clinical category, staging, or the molecular property of the BC-affected women. Testing panels of high- and moderate-risk genes in more than 60,000 affected women revealed substantial differences in clinical subtypes and associated genetic risk [72]. For example, the highest risk for ER-positive BC is associated with *ATM*, *CDH1*, and *CHEK2*, whereas *BARD1*, *BRCA1*, *BRCA2*, *PALB2*, *RAD51C*, and *RAD51D* confer a higher risk for ER-negative BC [73]. Rare pathogenic coding variants in these genes further contribute to overall BC risk [12,72].

In addition, there are more specialized cohorts of BC survivors that develop secondary primary cancers [74], BC patients that were exposed to radiation [75], and more. Such stratification is limited using population studies, as in retrospective biobanks. The contribution of rare variants from exome sequencing was not explicitly addressed in this study. As expected, rare variants are often below statistical significance [76]. Gene-based collapsing models for rare variants in UKB validated the significance of a small set of high BC risk (*BRCA1*, *BRCA2*, *PTEN*, *PALB2*) [72]. Recent large-scale analyses testing the impact of protein-truncating variants (LoF mutations) confirmed the known high-penetrance genes (*ATM*, *BRCA1*, *BRCA2*, *CHEK2*, *PALB2*), with only *MAP3K1* reaching exome-wide significance (Table 3). Other candidates, including *CDKN2A* and *BARD1*, did not reach statistical significance.

Overall, the rare variants’ impact on coding pathogenic variants appears modest, suggesting that even the combination of rare and common variants leaves a substantial fraction of genetic susceptibility unexplained [77]. While rare variants (<0.01% in the population) may have a limited contribution to screening efforts, they can benefit selected sub-populations. For example, *RECQL* is a DNA helicase that acts in preventing double-strand breaks. *RECQL* was recognized as a new BC susceptibility gene among Polish and Quebec populations, where each population is signified by a unique rare loss of function (LoF) variant [78]. Other examples of rare and ultra-rare LoF variants substantiate the importance of DNA repair mechanisms in the predisposition to BC [79]. Moreover, among BC-affected families from restricted populations, rare missense variants expose understudied cellular mechanisms that increase BC risk [53].

There are a few directions that can advance the clinical relevance of our findings. Our unified pipeline narrows the BC predisposition landscape to 38 high-confidence genes, refer to core BC gene set (Table 3). Eleven of the core BC genes already carry convincing epidemiological evidence for BC risk (binomial enrichment *p* = 1.47 × 10^−3^), among them four (*ATM*, *BRCA2*, *CHEK2*, *PALB2*) are currently interrogated by hereditary BC panels (Invitae, Myriad MyRisk, Table 3). The remaining genes represent actionable candidates for panel expansion, especially with supportive orthogonal validation (e.g., rare-variant, burden tests). The incorporation of genetic refined signal to the polygenic risk scores (PRS) can lead to improved personalized treatment on the BC field. Based on a large collection of studies, >170 common susceptibility loci were compiled whose individual effects are small but the combined PRS showed its value in stratifying women into subtype-specific scores. The reported PRS [80] showed that the higher 1% women at risk displayed 4.4-and 2.8-fold higher risk relative to the average women for ER-positive and ER-negative BC, respectively. We propose that incorporating the additional overlooked genes is likely to improve clinical utility and further boost the clinical utility and the development of personal cellular functional assays.

The initial PWAS analysis restricted to Caucasians [34] yielded only three significant genes. Yet the contribution of high- and medium-penetrance loci to breast-cancer risk varies markedly by ancestry: *ATM* shows limited relevance in Asians, founder *BRCA2* mutations greatly elevate risk in Ashkenazi Jews, pathogenic *BRCA2* and *PALB2* variants are more common in Black women, and *CHEK2* frequencies are lower in both Black and Asian populations. Expanding the cohort to all UKB participants and updating case numbers from 10.6k to 17.0k increased the discovery to eight significant genes, five of which are included in the BC core list with multiple lines of evidence (Table 3). PWAS that was expanded and included non-European population identified several new genes in female group (*MTMR11*, *CRLF3*, *FKBP5*). The additional genes that uncovered recessive associations (*TSC22D3*, *CHST7*, *C4BPB*) were previously unreported for familial BC, offering promising new candidates for clinical follow-up (Supplementary Figure S4).

This study carries several inherent limitations. First, collapsing methods such as PWAS and OT are restricted to protein-coding genes, so the contribution of non-coding RNAs, especially lncRNAs, to BC predisposition remains largely unexplored [81,82]. Second, cohort definitions were inconsistent. Specifically, the FG excluded participants with any non-breast malignancy (Supplementary Figure S2), whereas TWAS and PWAS did not. Some GWAS that contributed to OT collection relied on self-reporting. Another issue is that for UKB data field such as ICD-10 Z85.3 (“personal history of breast cancer”) may be wrongly added to the control group; therefore, the genetic signal might be diluted. These differences in ascertainment (i.e., cancer registry, ICD-10, Phecode, self-report) likely attenuated risk estimates. Finally, the sample was overwhelmingly Caucasian (Finns, other Europeans), with only modest numbers of Asian participants from MVP or UKB. The reduced power of the MVP African-ancestry subset, driven by smaller sample size, greater allelic heterogeneity, and lower imputation accuracy, highlights the contemporary challenges in studying non-European populations. Under these conditions, a lack of replication or bidirectionality in effect estimates is expected (Figure 4) and should not be inferred as biological divergence. Expanding well-powered African-ancestry resources will be critical for improving cross-ancestry inference in future work. Incorporating emerging biobanks such as All of Us and specialized national data from Mexico, Japan, and China will broaden the allelic spectrum, refine risk predictions, and provide evolutionary insight into BC susceptibility.

## 5. Conclusions

This study refines the landscape of heritable BC predisposition by applying a conservative, gene-centric framework that reduces false positives and emphasizes coherence across ExPheWAS, GWAS, TWAS, and PWAS. We defined a core set of 38 high-confidence susceptibility genes, including both established drivers and novel candidates, with several supported by recessive inheritance patterns. While our analyses are primarily based on European-ancestry cohorts and mostly focused on protein-coding genes, this framework improves mechanistic interpretability and provides a robust foundation for future studies. These results demonstrate the value of integrating multiple complementary gene-based approaches to prioritize high-confidence BC predisposition genes and highlight pathways warranting further investigation across diverse populations.

## Figures and Tables

**Table 1:** Top genes that were signified by stronger evidence beyond genetic association.

Gene	Main Feature/Function	Relevance to BC Predisposition	Role, [Penet.] ^a^	Population ^b^
CCNE1	Cell cycle: G1–S transition	Amplified in aggressive BC	BC driver	No
C11orf65	Mitochondrial-related gene	?	?	No
PTPN11	Phosphatase in RAS/MAPK signaling	Somatic mutations in BC tumors	?	No
RAD51B	DNA repair, homologous recombination	Low-penetrance gene	[Low]	European and Asian
BRCA2	DNA repair, homologous recombination	Strong association, hereditary BC	[High]	Ashkenazi Jews, Icelandic, French Canadian
DNMT3A	DNA methylation, epigenetic regulation	Common in blood cancers	?	No
ATM	DNA damage response; cell cycle checkpoints	Moderate-risk gene	[High-Mod]	European
ERBB4	EGFR family TK receptor	?	?	No
PALB2	BRCA2 binding partner; DNA repair	Strongly linked to hereditary BC	[High-Mod]	Finnish, French Canadian, Polish
ESR1	Estrogen receptor; hormone signaling	Common in ER-positive BC	BC driver	No

^a^ Penet., gene penetrance category. Mod, moderate; ?, no consensus for the role of penetrance in BC context. ^b^ Population specificity with founder variants.

**Table 2:** Enrichment test (FUMA-GWAS) for genes signified by markedly reduced OT global scores relative to their GA score.

GeneSet	N (n) ^a^	Adj. *p*-Value	Genes
BC	184 (16)	1.85 × 10^−25^	**PEX14**, **WDR43**, DLX2, CDCA7, NEK10, MRPS30, **CCDC170**, LMX1B, **DNAJC1**, **ZNF365,** ZMIZ1, FAR2, PAX9, **CCDC88C**, **TOX3**, **FTO**
BC (ER−)	25 (5)	6.98 × 10^−8^	**PEX14**, **WDR43**, **ZNF365**, **TOX3**, **FTO**
Breast size	59 (5)	4.29 × 10^−6^	**PEX14**, **CCDC170**, **ZNF365**, CCDC91, **FTO**
BC	17 (3)	6.31 × 10^−4^	**DNAJC1**, **CCDC88C**, STXBP4

^a^ N, the size of the genes listed as significant by any GWAS gene set; (n) is the number of genes from the input set used for the enrichment by FUMA-GWAS Gene2Func protocol. In bold, genes that are enriched in at least two of the four listed enriched independent GWAS gene sets.

**Table 3:** List of 45 candidate genes (included 38 core set) identified by a unification of results from large-scale association studies (GWAS, TWAS, and PWAS).

Gene Symbol	Shared Evidence ^a^	FG Coding #Var (V), #Ph ^b^	FG CS ^c^ ER+, ER−	Shared Driver ^d^	Clinial Panel ^e^	BC Predisp. Literature ^f^	Broad Functional Classification
ADAM29	a	-	ER+	-		No	Proteolysis/Cell adhesion
ALS2CR12	e	V1, Ph1	ER−	-		No	Cell projection
APOBEC3A	e	-	ER+	-		No	DNA/RNA editing
ARHGEF5 *	b	-		-		No	Rho GTPase signaling
ATM	a	V1, Ph2	ER+	-	I, M	Yes	DNA damage repair
BRCA2	c,f	V2, Ph2	ER+	TSG	I, M	Yes	DNA repair (homologous recombination)
CASP8	c	V1, Ph2		ONC		Yes	Apoptosis regulation
CCDC170	g,i	V2, Ph6		-		Yes	Structural protein (CC domain)
CCDC88C *	b	-		-		No	Cell migration signaling
CCDC91	f	-		-		No	Vesicle trafficking
CDKN2A	c	-		TSG		Yes	Cell cycle control
CDKN2B *	b	-		-		No	Cell cycle control
CHEK2	c,d,g	V3, Ph3	Shared	TGS	I, M	Yes	DNA damage checkpoint
DIRC3	e	-	ER+	-		Yes	Non-coding RNA regulator
DNAJC1	a	-	ER+	-		No	Protein folding (HSP40 chaperone)
EBF1	a	-		-		No	Transcription regulation
ESR1	a,i	-	ER+	-		Yes	Nuclear hormone receptor
EXO1	a	V1, Ph2	ER+	-		Yes#	DNA repair/recombination
FGFR2	a,i	-	Shared	-		Yes	Receptor tyrosine kinase/Signaling
FOXP1	c	-		TSG		No	Transcription regulation
FTO	a	-	Shared	-		No	RNA demethylase/Epigenetics
HNF4G *	b	-		-		No	Transcription regulation
LMX1B	a	-		-		No	Transcription regulation
LSP1	a,g	V3, Ph3	ER+	-		Yes	Actin cytoskeleton regulation
MAP3K1	b,c,i	V1, Ph2		TGS		Yes	MAPK signaling pathway
MLLT10	h	-		-		No	Chromatin remodeling/Transcription
MRPS30	b,g,i	V1, Ph4		-		No	Mitochondrial translation
NEK10	b,g,i	V2, Ph2		-		Yes	Cell cycle kinase
PAX9	a,i	-	ER+	-		No	Transcription regulation
PALB2	a	V1, Ph3	Shared	-	I, M	Yes	DNA repair (homologous recombination)
PEX14	d	-		-		No	Peroxisomal membrane transport
POU5F1B	a	V1, Ph2	ER+	-		No	Transcription regulation
RANBP9	d	-	ER+	-		No	Protein scaffolding
SETBP1	a	-		-		No	Transcription regulation
SLC4A7	e,i	-	ER+	-		Yes	Ion transport
SMC2 *	b	-		-		No	Chromosome condensation
STXBP4	d	V2, Ph2		-		No	Vesicular transport
TBX3	c	-		TSG		Yes	Transcription regulation
TCF7L2 *	b	-		-		No	Transcription regulation
TLR1 *	b	-		-		No	Innate immune receptor
TNS1	a	-		-		No	Focal adhesion signaling
TOX3	b,i	-	ER−	-		Yes	Transcription regulation
ZFP36L1	c,f	-		TSG		No	mRNA decay/Post-transcription
ZMIZ1	a	-	ER+	-		Yes	Transcription coactivation
ZNF365	a	V1, Ph2	Shared	-		Yes	Transcription/DNA repair

^a^ Evidence based on compilation of multiple large-scale results from this study. We merged a list of 45 associated genes. Evidence based on the shared genes of FG11, FG12, OT (at GA scores > 0.5 and >0.75), TWAS, and PWAS. The evidence source, labeled a–h is listed in Figure 6C. Evidence i is derived from ExPheWAS resource with q-value < 5 × 10^−7^ (Supplementary Table S9). There were 13 genes that are supported by multiple evidence sources (darker background, framed). Evidence b (OT > 0.75 and TWAS, labeled b) without further support is considered unreliable marked with *. ^b^ Var, variant, Ph, phenotypes related to breast cancer (including BC for ER negative, ER positive, disorder of breast, and more, See Supplemental Table S10). ^c^ CS, credible set, the ER+ and ER− are genes that were not detected by the broader phenotype and were either shared, or unique to BC subtypes. The genes that were identified in BC and also in the specific subtypes are indicated by the subtype. ^d^ TSG, ONC refers to tumor suppressor and oncogene, respectively. According to Figure 2B. ^e^ Clinical Invitae (I) or Myriad MyRisk (M) hereditary BC panels. ^f^ Yes and No refer to available literature support and to previous knowledge. #, mixed support in the literature. Gray, genes to be excluded from the Core BC predisposition genes.

## Data Availability

All results are available in the Supplementary Tables S1–S10. The computational pipeline for processing the UKB data used in this work is an open-source project available at https://github.com/nadavbra/ukbb_parser and for the FIRM model https://github.com/nadavbra/firm, accessed on 1 August 2025. The PWAS code and technical details are available in https://github.com/nadavbra/pwas. PWAS analysis on Europeans is presented in PWAS Hub https://pwas.huji.ac.il/ (ver2.0).

## Supplementary Materials

The following supporting information can be downloaded at: https://www.mdpi.com/article/10.3390/cancers17243969/s1, Table S1: Open Targets (OT) list of GA scores along with supporting evidence. Table S2: Classical GWAS for ICD10 C50 (Phecode 117.4) using all UKB for female cohort. Table S3. Coding gene GWAS (cgGWAS) from all females in the UKB cohort, *p*-value < 1.0 × 10^−5^. Table S4: FG (freeze 12) for BC using meta-analysis with 137 associated loci. Table S5: PWAS results for BC Phecode 174.11/ICD-10: C50 Europeans and Global. Table S6: List of C3_Breast_EXALLC from FG freeze 11 (FG11) credible set (CS). Table S7: List of C3_Breast_EXALLC from FG freeze 12 (FG12) credible set (CS). Table S8: List of 79 loci from TWAS and associated statistics based on GWAS eQTLs. Table S9: List of EXPheWAS (174.11) with corrected *p*-values for multiple phenotypes and genes. Table S10: Summary statistics of C3_Breast_(ERPLUS and ERNEG)_EXALLC from FG12. Figure S1. Mapping of Phecode with respect to ICD-10 (and ICD-9). (A) Circular presentation of PP174.11 that match C50 in ICD-10. The analysis performed on Phecode 174.11 (yellow) that covers the ICD-10 C50 (dark green). The G:C50 signs that the group includes children codes (shown in an external circles). (B) The same analysis according to a tree-like presentation. We focus on breast cancer of females (BC [F]) and have not included the BC of males (174.2). We show full names some of the subtree labels for ICD-9. Figure S2. Upset plot for FG12 for BC with 24,978 individuals. The plot lists the number of cases (with intersection) when combining different types of data source for breast cancer. The plot also marks the excluding sets of all other cancer. The BC phenotype title is shown. The labels on the left refer to the datasets used (and the number of individuals). The lines and dots show which datasets contribute to each intersection. Figure S3. Lavaa representation for Chr22:43810601-C. The nearest gene is TTC28. The dotted horizontal line indicates the genome-wide significance threshold (*p*-value 5.0 × 10^−8^). Negative beta values (left side, gray background) indicate a protective effect, or a decrease in risk. Significant phenotypes are listed along with the number of cases. Only significant phenotypes are labelled. The size of each bubble corresponds to the number of cases for the specific trait. This variant is associated with an increased risk for BC, thyroid cancer, kidney cancer, and hematologic malignancies. The multiple significant associations with increased risk imply a pleiotropic effect on malignancies. Figure S4. Histogram of the significant association signals (with FDR < 0.05) for candidate BC ICD10:C50 from both females and males for susceptibility genes. Bars show the −log10(*p*-value) observed for each gene under either dominant (yellow) or recessive (gray) models. Genes surpassing this threshold are labeled and ordered alphabetically. Rec, genes that are signified by a recessive inheritance. Figure S5. Venn diagram of the credible variants set (CS) by their gene symbols from three cohorts in FG12. All variants that failed mapping (marked as N.A.) were removed from the analysis. The list covers the BC-all (64 mapped, 58 unique genes). Another 11 and 39 (36 unique) genes derived from ER negative and ER positive cohorts, respectively. The genes that are shared and those that specify the BC subtypes are listed. The gene list is available in Supplemental Table S10.

## Abbreviations

The following abbreviations are used in this manuscript:

BC	breast cancer
CS	credible set
eQTL	expression quantitative trait locus
FG	FinnGen
GWAS	genome wide association study
ICD10	International Statistical Classification of Diseases and Related Health Problems,10th Revision
LD	linkage disequilibrium
LOF	loss of function
lncRNA	long non-coding RNA
MAF	minor allele frequency
MVP	million veteran program
ONC	oncogene
OR	odds ratio
OT	open targets
PheWAS	phenome wide association study
PRS	polygenic risk score
PWAS	proteome wide association study
TSG	tumor suppressor gene
TWAS	transcriptome wide association study
UKB	UK biobank
